# 739 Epidemiology of Accidental Burn Injuries: National Trends in Hospitalizations and Emergency Department Visits (2017-2022)

**DOI:** 10.1093/jbcr/irae036.282

**Published:** 2024-04-17

**Authors:** Darby Little, Stephanie A Mason

**Affiliations:** University of Toronto, Toronto, ON; Sunnybrook Health Sciences Centre, Toronto, ON; University of Toronto, Toronto, ON; Sunnybrook Health Sciences Centre, Toronto, ON

## Abstract

**Introduction:**

Understanding the evolving epidemiological trends of burns is essential for informing prevention strategies and healthcare resource allocation. While previous studies have described these trends within specific provinces, a comprehensive analysis of country-wide data is lacking.

**Methods:**

We conducted a national retrospective population-based study of all burn-related emergency department (ED) visits and hospitalizations over a 5-year period (2017-2022). Data were extracted from a national repository of health administrative data. Injuries due to assault and suicide attempt were not included.

**Results:**

Over a 5-year period there were 13,795 burn-related hospitalizations. There were four burn injury mechanisms identified in the database, from least to most common being related to electric current, explosion, fire and flame, and hot substances. The incidence of burns requiring hospitalization in the country remained constant over the 5-year period at 0.007%, though the absolute number of admissions generally increased over time. ED visit data were only available for 6 provinces in which there has been an overall decline in burn-related ED visits. The overall incidence of burns presenting to the ED decreased from 0.13% to 0.12% over the study period. Furthermore, the proportion of ED visits requiring hospitalization has increased over time from 5.4% to 6.4%.

**Conclusions:**

In summary, this national population-based study of burn injuries from 2017-2022 has demonstrated that while the incidence of burn-related ED visits in several provinces has decreased, the national incidence of burn-related hospitalizations in the country has remained stable. These findings shed light on the changing landscape of burn injuries and underscore the need for continued monitoring and targeted prevention strategies.

**Applicability of Research to Practice:**

This study offers important insights into the epidemiology of burn injuries from 2017-2022 and provides a foundation for evidence-based decision-making in burn injury resource allocation.

